# Career-Choice Regret Among Medical Students in the United Kingdom: An Exploratory Mixed-Methods Study of Occupational and Organizational Perceptions

**DOI:** 10.7759/cureus.118523

**Published:** 2026-10-24

**Authors:** Alexia Davy

**Affiliations:** 1 Acute Medicine, University Hospitals of Leicester NHS Trust, Leicester, GBR

**Keywords:** career choice, career-choice regret, career satisfaction, medical education, medical students, national health service, occupational regret, organisational regret, physician workforce, postgraduate medical training

## Abstract

Background: Career-choice regret has been reported among medical students internationally, but less is known about how career-choice responses coexist with perceptions of medicine as a profession and the National Health Service (NHS) as a potential employer among United Kingdom (UK) medical students. This study explored career-choice responses and occupational and organizational perceptions among UK medical students.

Methods: An exploratory cross-sectional mixed-methods online survey was completed by 31 medical students from 22 UK universities who were recruited through convenience and snowball sampling. The primary outcome was participants’ response to whether they would choose medicine again. Occupational and organizational perceptions were assessed using two newly developed, unvalidated 10-item composite perception measures. Participants also rated the importance of and expected satisfaction with career characteristics. Quantitative data were analyzed descriptively, and free-text responses from 18 participants were analyzed thematically.

Results: Of 31 participants, 14 (45.2%) reported that they would choose medicine again, 10 (32.3%) were unsure, and 7 (22.6%) reported that they would not. Mean occupational and organizational composite scores were 25.23 (SD 6.34) and 38.97 (SD 8.20), respectively. Item-level responses suggested positive views of medicine as a profession alongside negative perceptions of several aspects of NHS employment. The largest gaps were between the importance attached to career characteristics and expected satisfaction related to work location, access to specialty training, work-life balance, and the work environment. Twenty-seven participants (87.1%) considered regret and dissatisfaction among medical students to be a common problem. Free-text responses highlighted concerns about medical education and postgraduate training, work-life balance, future career prospects, financial and working conditions, and feeling undervalued by the NHS. Other responses conveyed continued enthusiasm for medicine despite concerns about the organizational context.

Conclusions: In this small, self-selected sample, positive perceptions of medicine as a profession coexisted with negative perceptions of the NHS as an organization, while fewer than half of participants reported that they would choose medicine again. These exploratory findings describe career-choice responses and occupational and organizational perceptions but do not establish a causal relationship between organizational dissatisfaction and stated willingness to choose medicine again or predict future career decisions. Larger studies using more diverse samples, validated measures, and longitudinal follow-up are needed to investigate how these perceptions relate to career choices over time.

## Introduction

Choosing a career in medicine is a major life decision, requiring substantial investment of time, education, and personal resources [1,2]. Medical students make this decision before experiencing the full realities of working as a doctor and may therefore have limited insight into the demands of medical education and their future careers. Medical careers can involve demanding workloads, emotional pressures, professional responsibility, and challenges to work-life balance, which may differ from students’ expectations when entering medical education [3]. A mismatch between expectations and perceived reality may therefore contribute to dissatisfaction with or regret about the decision to pursue medicine. Understanding how students evaluate this decision is important, particularly given the implications of workforce satisfaction and retention for the sustainability of the National Health Service (NHS) [4,5].

Career-choice regret describes negative feelings arising from the belief that an alternative career decision would have resulted in a more desirable outcome [6]. It is related to, but distinct from, career dissatisfaction, as an individual may be dissatisfied with particular aspects of their career without regretting the decision to enter it. Conversely, an individual may question whether choosing a career was the right decision while continuing to value aspects of their occupation or experience positive aspects of their work. This distinction is particularly relevant to medicine, where dissatisfaction may arise from the conditions under which doctors practice rather than from medicine itself as an occupation. Research among UK doctors has identified working conditions, workload, remuneration, work-life balance, training, and the wider NHS environment as factors that may influence decisions about remaining in UK medicine [7]. Regret may therefore reflect different aspects of a career decision, including the occupation chosen and the organizational context in which that occupation is pursued.

Previous research has identified career-choice regret among medical students in several countries, with reported experiences varying across populations and stages of training [8-11]. Among medical students, career regret has been associated with factors including burnout, the learning environment, and health-related quality of life [9,10]. However, much of the wider literature examining dissatisfaction, retention, and career decision-making concerns qualified doctors, whose experiences of employment may differ from those of students who are still developing expectations of their future careers. Research among UK doctors in the early stages of their careers has demonstrated that workplace experiences, working practices, career structures, and perceptions of future prospects can influence career decisions [12]. Findings from different countries, healthcare systems, and stages of training may not therefore be directly applicable to contemporary UK medical students. Further research is needed to examine how medical students in the UK evaluate their decision to pursue medicine and the factors associated with regret or uncertainty about that decision.

Career regret may also arise from dissatisfaction with different aspects of the context in which an occupation is pursued [6]. One medical student may question becoming a doctor because of the nature of medical work itself, while another may remain committed to medicine but develop negative perceptions of the organizational context in which doctors practice. This distinction may be particularly important in the United Kingdom (UK), where medical students undertake much of their clinical education within the NHS, and many subsequently begin their medical careers within the NHS. Through clinical placements and interactions with healthcare professionals, students are exposed to aspects of NHS working environments and may develop perceptions of working conditions, professional support, resources, workload, remuneration, and work-life balance. Experiences of workplaces and working practices have been shown to influence UK doctors’ perceptions of their careers and subsequent career decisions [12], while dissatisfaction with the NHS environment has also been identified among doctors who subsequently leave UK medicine [7,13]. These perceptions may influence how attractive a medical career appears and shape expectations of future employment. However, comparatively little research has examined whether regret relates differently to perceptions of medicine as an occupation and the organizational context in which doctors practice.

Students may also evaluate a medical career according to the extent to which it is expected to provide characteristics that they consider important. Factors such as work-life balance, the work environment, flexibility, career progression, remuneration, and control over working location may influence perceptions of career attractiveness and satisfaction [14,15]. However, the characteristics students value most highly may not be those they expect medicine to provide most effectively. For example, a student may consider work-life balance highly important while expecting a medical career to provide relatively poor work-life balance. This creates a discrepancy between the importance assigned to a career characteristic and the satisfaction expected from it, representing a potential mismatch between career priorities and anticipated career experiences. Such mismatches may be relevant to career-choice regret because regret can arise when the perceived outcome of a decision falls short of what an individual values or expects to obtain from their career [6]. Examining these importance-satisfaction discrepancies may therefore provide a more nuanced understanding of how students evaluate their decision to pursue medicine.

This exploratory mixed-methods study aimed primarily to describe UK medical students’ stated willingness to choose medicine again, assessed using a single career-choice question. Secondary exploratory objectives were to describe perceptions of medicine as an occupation and NHS employment as a potential workplace, using the newly developed and unvalidated Occupational Perceptions Scale (OccPS) and Organizational Perceptions Scale (OrgPS), respectively. The study also examined the importance participants attached to selected career characteristics and their expected satisfaction with these characteristics. Free-text responses were used to contextualize the descriptive quantitative findings and explore participants’ perspectives on career choice, medical training, and anticipated working conditions. The study was designed to describe patterns within the respondent sample and not to test hypotheses, establish causal or predictive relationships, or make population-level estimates.

## Materials and methods

Study design and participants

An exploratory, cross-sectional mixed-methods survey was conducted to examine UK medical students’ stated willingness to choose medicine again, perceptions of medicine as an occupation, perceptions of the NHS as a potential employer, and expectations regarding future working conditions and career characteristics. The study was designed to describe these perceptions within the respondent sample; it was not designed to estimate their prevalence among all UK medical students or to establish causal relationships.

The target population comprised medical students enrolled at UK medical schools during the 2023-2024 academic year. Students from all years of study and course types were eligible to participate, including those undertaking intercalated degrees or taking time out from their studies. Participants were recruited using convenience sampling supplemented by snowball sampling.

Recruitment and data collection

Data were collected using an anonymous online questionnaire hosted on Jisc Online Surveys for six weeks between July and August 2024 (Appendix A). The survey was distributed through medical student networks, student groups, Reddit, social media, and the researcher's academic networks. Participants were also encouraged to share the survey with eligible colleagues.

To reduce the potential for response bias associated with the topic under investigation, recruitment materials did not explicitly frame the study solely as an investigation of career regret. No personally identifiable information was collected.

Sample size

The planned sample size was determined a priori using an estimated UK medical student population of approximately 41,000 students, as reported by Feng et al. [16]. For the quantitative component, a target of 96 participants was calculated using a 95% confidence level, a 10% margin of error, and a conservative assumed population proportion of 50%, with a finite-population adjustment. This target was intended to guide an exploratory survey rather than support population-level generalizability or definitive hypothesis testing. For the qualitative component, an initial target of approximately 20 participants was proposed to provide sufficient depth of free-text responses and enable exploration of recurring themes. These targets were pragmatic and appropriate to the mixed-methods exploratory design rather than based on formal power calculations.

Recruitment used convenience and snowball sampling, and the questionnaire remained open for six weeks. The final sample comprised 31 respondents from 22 UK universities, which was smaller than the planned sample of 96. As the sample was small and recruited using nonprobability methods, the sample-size calculation should not be interpreted as establishing representativeness or population-level precision. All reported frequencies and percentages are descriptive summaries of the respondents and are not population estimates for UK medical students.

A total of 34 questionnaire records were initiated. Three incomplete questionnaires were excluded before analysis because they had not been completed and contained insufficient data for the planned analyses. The final analytic sample therefore comprised 31 completed questionnaires. No data from incomplete questionnaires were included in the quantitative or qualitative analyses.

Ethical considerations

The study was conducted in accordance with ethical guidelines for research involving human participants and received ethical approval from the University of Leicester Ethics Committee.

Before accessing the questionnaire, participants were provided with a participant information sheet describing the purpose of the study, potential risks and benefits of participation, and their right to withdraw. Participants were required to confirm that they had read and understood the information provided and consented to participate before proceeding to the first survey question. No personally identifiable information was collected, and responses were therefore anonymous. Study data were stored securely and were accessible only to the researcher.

Because discussion of career regret and dissatisfaction had the potential to cause distress, participants were provided with information about relevant mental health support resources following completion of the questionnaire and were encouraged to seek support if needed.

Questionnaire and measures

The questionnaire collected demographic information, occupational and organizational perceptions, expectations concerning career characteristics, responses to a single career-choice question, and free-text responses concerning participants’ experiences and views. The single career-choice question was the primary outcome. Occupational and organizational items were analyzed as newly developed, unvalidated exploratory measures of perceptions; they were not treated as validated measures of career regret (Appendix B).

Questionnaire Development

The questionnaire was developed specifically for this study. Items assessing perceptions of medicine as an occupation were informed by previous literature on career-choice regret, occupational regret, and medical students’ career perceptions [8,10,14,17]. Items assessing perceptions of the NHS as an organization were developed with reference to literature concerning doctors’ working conditions, workforce retention, and reasons for leaving UK medicine [5,7,13].

The occupational and organizational items were grouped into two newly developed composite measures: the OccPS and OrgPS. These measures were developed for exploratory use in the present study and had not undergone formal psychometric development or external validation. Although the abbreviations are retained for consistency, the measures should be understood as assessing occupational and organizational perceptions rather than as validated measures or dimensions of career-choice regret. The OccPS and OrgPS were included as secondary exploratory measures to provide contextual information on occupational and organizational perceptions alongside the primary career-choice question. They were not intended to confirm a structure of career regret or to establish predictors of future career decisions.

The grouping of items into the OccPS and OrgPS, the reverse-scoring rules, and the direction of total-score interpretation were specified before quantitative analysis, based on the occupational and organizational domains of the questionnaire. These were nevertheless exploratory, author-developed composite measures rather than validated scales.

Internal consistency of the composite measures was assessed using Cronbach’s alpha. This analysis provides evidence only of internal consistency within the present sample and does not establish construct validity, test-retest reliability, or reliability across settings and populations. The composite scores were therefore used for exploratory descriptive purposes and interpreted cautiously. Because the measures contained different item content, their mean scores were not treated as directly comparable.

Demographic Characteristics

Participants were asked about age, gender, medical course type, year of study, previous educational qualifications, whether they had taken time out from medical education, and whether medicine had been their first-choice career.

Occupational Perceptions

Occupational perceptions were assessed using 10 five-point Likert scale items addressing participants' views of medicine as a profession. Items assessed perceived suitability for medicine, enthusiasm for the profession, perceived ability to succeed, the effect of studying medicine on personal life, respect for the profession, career opportunities, the value of a medical degree, career choice, and willingness to recommend medicine to others.

The items were informed by measures used in previous studies of career regret and career perceptions among medical students and professionals [8,10,17]. Responses were coded from 1 to 5, with scoring arranged such that higher scores represented more negative occupational perceptions. The 10 items were summed to produce an exploratory OccPS score, with possible scores ranging from 10 to 50.

Organizational Perceptions

Perceptions of the NHS as a potential employer were assessed using 10 five-point Likert scale items designed to parallel the occupational measure. Item content was informed by previous UK literature examining doctors’ experiences of NHS employment, including organizational support, working conditions, career opportunities, perceived value, workload, work-life balance, and intentions to remain in medicine [7,13,18]. Items addressed perceived respect for doctors working in the NHS, career opportunities, organizational support, perceived value of employees, career choice, ability to succeed, concerns regarding resources, willingness to work in the NHS, anticipated effects on personal life, and willingness to recommend NHS employment to others.

Responses were coded from 1 to 5, with higher scores representing more negative perceptions of the NHS. The 10 items were summed to produce an exploratory OrgPS score, with possible scores ranging from 10 to 50.

Career Characteristics

Participants rated 16 characteristics of a medical career according to both their importance when considering a career and their expected satisfaction with each characteristic when working as a doctor. The characteristics comprised 12 nonmonetary factors and four monetary factors informed by previous research [5,13,14,16].

Nonmonetary factors included the ability to choose a working location, ease of entry into specialty training, work-life balance, work environment, workload, flexible working, job security, recognition and respect at work, professional autonomy, job benefits, length of specialty training, and societal perceptions of doctors. Monetary factors comprised pay during Foundation Years 1 and 2, costs associated with training, pay during specialty training, and pay at the consultant level.

Mean importance and satisfaction ratings were calculated for each characteristic. An importance-satisfaction discrepancy score was calculated by subtracting the mean satisfaction rating from the mean importance rating, with larger positive values indicating a greater gap between the importance attached to a factor and expected satisfaction with it.

Primary Outcome: Career-Choice Question

The primary outcome was participants’ response to a single career-choice question adapted from Tian et al., who assessed career-choice regret using the question, “If you could go back, would you choose to become a doctor again?” [19]. In the present study, the wording was modified to “If you could go back, would you still choose to become a doctor?” Participants selected “Yes,” “Unsure,” or “No.”

This item was used to describe respondents’ stated willingness to choose medicine again. A “No” response indicated that the respondent would make a different career choice if given the opportunity, whereas an “Unsure” response indicated career-choice uncertainty. Neither uncertainty nor negative occupational or organizational perceptions was treated as equivalent to career regret. As a single adapted item, this question was not treated as a validated measure of career-choice regret. Where comparisons were made, the three response groups were retained separately.

Quantitative analysis

Quantitative data were analyzed using IBM SPSS Statistics (IBM Corp., Armonk, NY, USA). Categorical variables, including responses to the primary career-choice question, were summarized using frequencies and percentages. All percentages describe the observed responses in this small convenience sample and were not interpreted as estimates of the wider UK medical student population. Continuous variables and composite perception scores were summarized using means and standard deviations.

OccPS and OrgPS scores were calculated by summing responses across their respective 10 items, and internal consistency was assessed using Cronbach’s alpha. As the measures were newly developed, unvalidated, and contained different item content, their scores were interpreted cautiously and were not treated as directly comparable measures of the same construct. Mean importance, satisfaction, and importance-satisfaction discrepancy scores were calculated for each of the 16 career characteristics.

Responses to the primary career-choice question were summarized across the three response categories. Occupational and organizational perception scores were described separately according to participants’ responses to this question and to the question concerning whether regret and dissatisfaction among medical students were perceived to be a common problem. Given the exploratory design and small sample size, no inferential statistical testing was undertaken; all subgroup comparisons were descriptive.

Missing Data

The analysis was restricted to completed questionnaires. Item-level analyses were conducted using available responses; denominators are reported where they differed from the full analytic sample of 31 respondents. No imputation was performed. Blank optional free-text responses were treated as indicating that the respondent did not contribute qualitative data to that question and were not imputed.

For the OccPS and OrgPS, composite scores were calculated only for respondents with complete responses to all 10 items within the relevant scale. Respondents with one or more missing component items were not assigned a score for that scale.

Qualitative analysis

The questionnaire included free-text questions asking respondents to explain their career-choice response, including reasons for uncertainty or for not choosing medicine again, and to suggest changes that might reduce dissatisfaction among medical students. All nonempty free-text responses were included in the qualitative analysis.

Free-text responses were analyzed using exploratory thematic analysis to provide contextual insight into respondents’ stated career-choice responses and occupational and organizational perceptions. Analysis was conducted by the lead researcher, who reviewed all responses repeatedly to become familiar with the dataset. Initial codes were generated from the content of the responses, focusing on expressed concerns, experiences, and proposed changes. Codes with similar meanings were then grouped into candidate themes, which were reviewed against the original responses and refined into broader themes.

The analysis was primarily data-driven and descriptive, as the brief free-text format did not permit the depth of exploration available through interviews. Themes were used to summarize recurring patterns in the respondent accounts rather than to estimate prevalence, establish causation, or generate findings generalizable to UK medical students. Quotations were selected to illustrate themes and were minimally edited only where necessary for readability; participant identifiers were retained to preserve anonymity.

A single response could contribute to more than one code or theme. Themes relating to reasons for career-choice uncertainty or not choosing medicine again were considered separately from themes relating to proposed solutions.

Because the analysis was conducted by one researcher, coding and theme development were not independently checked. The findings should therefore be interpreted as an exploratory interpretation of brief free-text responses.

At the time of the study, the lead researcher was a medical student and recognized that their prior interest in medical student career choice, together with awareness of concerns regarding medical training and NHS employment, could influence coding and interpretation. This shared educational context may have informed their understanding of participants’ accounts. To reduce this influence, themes were developed inductively from the content of the free-text responses, reviewed against the original dataset, and illustrated using representative quotations. Nevertheless, the analysis represents a single-researcher interpretation of the data.

Quantitative and qualitative findings were analyzed separately and integrated during interpretation and reporting. Quantitative findings described response patterns, perception scores, and importance-satisfaction discrepancies, while qualitative themes were used to contextualize these descriptive patterns. Integration was presented through a joint display table linking selected quantitative findings with qualitative themes and representative quotations. Qualitative findings were not used to test, validate, or establish causal relationships between quantitative measures and career-choice responses.

## Results

Participant characteristics

A total of 31 complete responses were received from students representing 22 UK universities. Fifteen participants (48.4%) identified as male, 15 (48.4%) as female, and one (3.2%) chose not to disclose their gender. Participants were aged between 18 and 66 years, with a mean age of 25 years (Table 1).

**Table 1:** Demographic breakdown of the study sample (N=31)

Characteristic		n	%
Gender	Male	15	48.4
	Female	15	48.4
	Prefer Not to Say	1	3.2
Course Type	Undergraduate – 5 Year	19	61.3
	Undergraduate – 6 Year	5	16.1
	Medicine With a Foundation Year	3	9.7
	Graduate Entry Medicine	4	12.9
Year of Study	Foundation (Year 0)	1	3.2
	Year 1	5	16.1
	Year 2	7	22.6
	Year 3	2	6.5
	Year 4	8	25.8
	Year 5	2	6.5
	Intercalation	3	9.7
	Time Out of Studies	3	9.7
Prior Qualifications	School Leaver	22	71.0
	Undergraduate Degree	6	19.4
	Postgraduate Degree	3	9.7
1^st^ Choice of Career	Yes	23	74.2
	No	8	25.8
Age	Mean (Range)	25 (18 - 66)	

Nineteen participants (61.3%) were enrolled in a standard five-year undergraduate medical course, five (16.1%) in a six-year undergraduate course, three (9.7%) in a medical course with a foundation year, and four (12.9%) were undertaking graduate-entry medicine. Participants were represented across all years of study, including students undertaking intercalation and those taking time out of their studies.

Twenty-two participants (71.0%) entered medicine directly from school, six (19.4%) had previously obtained an undergraduate degree, and three (9.7%) had obtained a postgraduate degree. Medicine was reported to have been the respondents' first-choice career by 23 participants (74.2%), while eight (25.8%) reported that it had not been their first choice.

Occupational perceptions

Participants generally expressed positive perceptions of medicine as an occupation, although several responses indicated substantial dissatisfaction with aspects of the career. Appendix C shows participant responses to the individual OccPS items.

Twenty-eight participants (90.3%) agreed or strongly agreed that medicine was a field that genuinely excited them, while the same proportion reported feeling a strong sense of empathy toward patients and their well-being. Twenty-five participants (80.6%) agreed or strongly agreed that they had the ability to succeed in medicine.

Perceptions were less positive regarding the consequences of studying medicine and willingness to recommend the profession. Eighteen participants (58.1%) agreed or strongly agreed that they sometimes wished they had pursued a different profession, while 20 (64.5%) agreed or strongly agreed that studying medicine had negatively affected their personal lives. Only nine participants (29.0%) agreed or strongly agreed that they would recommend a career in medicine to a friend.

Medicine continued to be perceived as a respected and valuable profession by many participants. Eighteen participants (58.1%) agreed or strongly agreed that medicine was a well-respected profession, while 25 (80.6%) agreed or strongly agreed that a medical degree was a highly valued qualification. Twenty participants (64.5%) agreed or strongly agreed that medicine provided good career opportunities.

The mean OccPS score was 25.23 (SD 6.34). Female participants had a mean score of 26.07 (SD 5.24), compared with 25.27 (SD 6.73) among male participants. One participant did not disclose their gender and was not included in the gender-specific summaries. Cronbach's alpha for the 10-item OccPS was 0.761 (Table 2).

**Table 2:** Occupational and organizational perception scores among participants (N=31) OccPS, Occupational Perceptions Scale; OrgPS, Organizational Perceptions Scale

Measure	Mean ± SD	Cronbach's α
OccPS	25.23 ± 6.34	0.761
OrgPS	38.97 ± 8.20	0.910

Organizational perceptions of the NHS

Item-level responses to questions about NHS employment reflected substantial concerns regarding several aspects of the organizational context. Appendix D shows participant responses to the individual OrgPS items.

Only one participant (3.2%) agreed that the NHS provided a supportive work environment, while 25 participants (80.6%) disagreed or strongly disagreed with this statement. Similarly, only one participant (3.2%) agreed that the NHS valued its employees, compared with 24 participants (77.4%) who strongly disagreed or disagreed with the statement.

Seven participants (22.6%) agreed or strongly agreed that working as a doctor in the NHS was a well-respected job, compared with 16 (51.6%) who disagreed or strongly disagreed. Similarly, only seven participants (22.6%) agreed or strongly agreed that the NHS provided good career opportunities, while 17 (54.8%) disagreed or strongly disagreed.

Fourteen participants (45.2%) agreed, and three (9.7%) strongly agreed that they had the ability to succeed as a doctor in the NHS. However, 28 participants (90.3%) agreed or strongly agreed that they worried about a lack of resources in the NHS.

Twenty participants (64.5%) agreed or strongly agreed that, given a choice, they would not work as a doctor in the NHS. Twenty-three participants (74.2%) agreed or strongly agreed that working in the NHS would negatively affect their personal lives. Only two participants (6.5%) agreed or strongly agreed that they would recommend working in the NHS to a friend.

The mean OrgPS score was 38.97 (SD 8.20). Female participants had a mean score of 40.67 (SD 6.66), compared with 39.00 (SD 6.97) among male participants. One participant did not disclose their gender and was not included in the gender-specific summaries. Cronbach's alpha for the 10-item OrgPS was 0.910 (Table 2).

Occupational and organizational perceptions

The results demonstrated a distinction between participants' perceptions of medicine itself and their perceptions of the NHS as the organizational context in which they expected to practice.

Although 90.3% (n=28) of participants agreed or strongly agreed that medicine was an exciting field and 80.6% (n=25) believed they had the ability to succeed in medicine, only 54.8% (n=17) agreed or strongly agreed that they had the ability to succeed as a doctor within the NHS.

Similarly, 64.5% (n=20) of participants reported that studying medicine had negatively affected their personal lives, while a larger proportion, 74.2% (n=23), expected working in the NHS to have a negative effect on their personal lives.

A marked difference was also observed in perceptions of respect and organizational value. While 58.1% (n=18) considered medicine itself to be a well-respected profession, only 22.6% (n=7) considered working as a doctor in the NHS to be a well-respected job. Likewise, 80.6% (n=25) considered a medical degree to be a highly valued qualification, whereas only 3.2% (n=1) agreed that the NHS valued its employees.

Item-level responses suggested that participants reported positive views of several aspects of medicine while also reporting concerns about NHS employment conditions. Because the OccPS and OrgPS differed in content and were unvalidated, their total mean scores were not directly compared.

Importance and expected satisfaction with career characteristics

Participants rated the importance of 16 career characteristics alongside their expected satisfaction with each factor. The largest importance-satisfaction discrepancies were observed for factors relating to career progression, geographical control, and working conditions (Table 3).

**Table 3:** Importance and expected satisfaction with career characteristics among participants (N=31) Importance and expected satisfaction were rated on five-point Likert scales, with 1 indicating low importance/satisfaction and 5 indicating high importance/satisfaction. Discrepancy was calculated as mean importance minus mean expected satisfaction; positive values indicate that expected satisfaction was lower than perceived importance. Note: Discrepancy scores were calculated from unrounded mean values and may therefore differ slightly from subtraction of the displayed rounded means.

		Mean Importance ± SD	Mean Expected Satisfaction ± SD	Discrepancy
Non-Monetary Factors	Ability to Choose Working Location	4.32 ± 1.44	1.65 ± 0.98	2.68
	Ease of Entry into Specialty Training	4.35 ± 0.80	1.84 ± 1.00	2.52
	Work-Life Balance	4.23 ± 0.84	1.84 ± 0.86	2.39
	Work Environment	4.19 ± 0.87	2.00 ± 0.89	2.19
	Workload	3.77 ± 0.99	1.87 ± 0.99	1.90
	Flexible Working	3.87 ± 1.06	2.03 ± 1.02	1.84
	Job Security	3.94 ± 0.96	2.68 ± 0.98	1.26
	Recognition and Respect at Work	3.65 ± 1.05	2.42 ± 1.23	1.23
	Professional Autonomy	3.74 ± 1.09	2.74 ± 1.09	1.00
	Job Benefits	3.16 ± 1.19	2.35 ± 1.28	0.81
	Length of Specialty Training	3.39 ± 1.33	2.58 ± 1.09	0.81
	Societal Perception of Doctors	2.42 ± 1.09	3.10 ± 1.11	-0.68
Monetary Factors	Pay During Foundation Years 1 and 2	4.00 ± 1.18	1.68 ± 0.94	2.32
	Costs Associated With Training	4.00 ± 1.06	1.77 ± 0.84	2.23
	Pay During Specialty Training	4.06 ± 0.81	2.10 ± 1.08	1.97
	Pay at Consultant Level	3.90 ± 1.14	2.74 ± 1.09	1.16

Among nonmonetary factors, ease of entry into specialty training had the highest mean importance rating, followed by ability to choose working location, work-life balance, and work environment. Expected satisfaction was lowest for ability to choose working location and ease of entry into specialty training, with importance-satisfaction gaps of 2.68 and 2.52, respectively.

Work-life balance also demonstrated a substantial discrepancy, with a mean importance rating of 4.23 and satisfaction rating of 1.84, giving a gap of 2.39. Work environment, workload, and flexible working had gaps of 2.19, 1.90, and 1.84, respectively.

Job security, recognition and respect at work, professional autonomy, job benefits, and length of specialty training demonstrated smaller positive discrepancies. Societal perception of doctors was the only factor for which expected satisfaction exceeded importance, with an importance rating of 2.42 and satisfaction rating of 3.10, producing a negative discrepancy of -0.68.

Among monetary factors, pay during specialty training was rated as the most important (4.06), followed by pay during Foundation Years 1 and 2 and costs associated with training, both rated 4.00. Expected satisfaction was lowest for Foundation Years 1 and 2 pay (1.68), followed by costs associated with training (1.77) and specialty training pay (2.10).

Foundation-level pay therefore demonstrated the largest monetary importance-satisfaction gap (2.32), followed by training costs (2.23) and specialty training pay (1.96). Consultant-level pay had the smallest monetary discrepancy, with an importance rating of 3.90 and satisfaction rating of 2.74, producing a gap of 1.16.

Overall career-choice response

When asked, “If you could go back, would you still choose to become a doctor?”, 14 participants (45.2%) answered yes, 10 (32.3%) were unsure, and seven (22.6%) answered no (Table 4). Among male participants, six (40.0%) would choose medicine again, six (40.0%) were unsure, and three (20.0%) would not choose medicine again. Among female participants, seven (46.7%) would choose medicine again, four (26.7%) were unsure, and four (26.7%) would not choose medicine again.

**Table 4:** Career-choice response and OccPS/OrgPS scores (N=31) OccPS, Occupational Perceptions Scale; OrgPS, Organizational Perceptions Scale

		n (%)	OccPS Mean ± SD	OrgPS Mean ± SD
If you could go back, would you still choose to become a doctor?	Yes	14 (45.2)	21.43 ± 6.00	36.43 ± 9.50
	Unsure	10 (32.3)	27.10 ± 4.70	39.70 ± 6.56
	No	7 (22.6)	30.14 ± 3.27	43.00 ± 4.07
Do you think that regret and dissatisfaction among medical students is a common problem?	Yes	27 (87.1)	26.11 ± 6.10	39.52 ± 8.09
	No	4 (12.9)	19.25 ± 3.03	35.25 ± 6.87

Mean occupational and organizational perception scores varied according to responses to the overall career-choice question. Participants who would choose medicine again had the lowest mean OccPS and OrgPS scores (21.43 and 36.43, respectively). Those who were unsure had higher mean scores of 27.10 for OccPS and 39.70 for OrgPS, while participants who would not choose medicine again had the highest scores, at 30.14 and 43.00, respectively.

A similar descriptive pattern was observed in relation to whether participants considered regret and dissatisfaction among medical students to be a common problem. Most respondents considered regret and dissatisfaction among medical students to be a common problem, with 87.1% (n=27) selecting this response.

Participants who considered it a common problem had mean OccPS and OrgPS scores of 26.11 and 39.52, respectively, compared with 19.25 and 35.25 among those who did not (Table 4).

Qualitative findings

Of the 31 completed questionnaires, 18 included at least one free-text response and were included in the qualitative analysis. Of these respondents, nine reported that they would still choose medicine again, four reported that they would not, and five were unsure. The free-text findings therefore reflect the views of this subgroup only and should not be assumed to represent the entire sample or UK medical students more broadly.

Themes were developed from the free-text responses to describe recurring concerns and suggestions within this subgroup. Among the nine respondents who reported that they would not choose medicine again or were unsure, six provided a substantive explanation in the free-text question asking why they felt this way. As a response could contribute to more than one theme, thematic categories were not mutually exclusive. Concerns about job satisfaction and future career prospects were identified in five of these six responses; concerns about medical education or postgraduate training were identified in four; and concerns about inadequate rewards or NHS employment conditions were identified in four.

A contrasting theme was identified among two of the nine respondents who stated that they would still choose medicine again: continued commitment to medicine alongside negative perceptions of NHS employment, training, or working conditions. Selected anonymized quotations are provided to illustrate the themes.

Table 5 integrates the principal descriptive quantitative patterns with the qualitative themes and selected representative quotations, including accounts that contrast continued commitment to medicine with dissatisfaction with NHS employment or training conditions.

**Table 5:** Integration of descriptive quantitative and qualitative findings Free-text responses were provided by 18 of 31 respondents. Thematic counts relating to reasons for uncertainty or choosing not to enter medicine use the six respondents who provided a substantive explanation; a response could contribute to more than one theme. OccPS and OrgPS subgroup patterns are descriptive only and do not establish statistical differences or causal relationships. OccPS, Occupational Perception Scores; OrgPS, Organizational Perception Scores; NHS, National Health Service

Descriptive Quantitative Finding	Qualitative Finding	Representative Quotation
In the full sample (n=31), 14 respondents (45.2%) reported “Yes,” 10 (32.3%) were unsure, and 7 (22.6%) reported “No” to the career-choice question.	Among the 18 respondents providing free text, six of the nine who were unsure or answered “No” provided a substantive explanation. Concerns about medical education or postgraduate training were identified in four of these six responses (4/6).	“The course I am currently studying lacks the clinical exposure that I would have desired, and I am no longer sure if I am interested in the realities of being a doctor.” (Participant 5)
The largest importance-satisfaction discrepancies concerned working location (2.68), entry into specialty training (2.52), work-life balance (2.39), and working environment (2.19).	Concerns about job satisfaction and future career prospects were identified in five of the six substantive explanations (5/6).	“I didn't realise how expensive, difficult, and competitive training beyond Foundation would be.” (Participant 9)
OccPS and OrgPS scores were summarized descriptively by career-choice response group (Table 4). No inferential testing was undertaken. As the measures assess different content and are newly developed and unvalidated, their scores should not be directly compared.	Contrasting accounts were identified: two of the nine respondents who reported that they would still choose medicine again also described negative perceptions of NHS employment, training, or working conditions.	“I love medicine, I love seeing patients and trying to help solve their problems. I just wish I had more control over my life.” (Participant 8)
Of the 31 respondents answering the relevant question, 27 (87.1%) considered regret or dissatisfaction among medical students to be a common problem.	12 respondents suggested at least one change. Pay or working conditions were raised in six (6/12) responses; training reform in five (5/12); healthcare-system or policy changes in four (4/12); and greater priority for United Kingdom graduates in training pathways in three (3/12).	“Better pay and working conditions. Striving for better autonomy over location choices - rather than being randomly allocated, have some say…” (Participant 17)

Medical Education and Postgraduate Medical Training

Some participants described dissatisfaction with the structure and delivery of medical education. Concerns included limited clinical exposure, the pace of teaching, and the perceived emphasis on progressing through large volumes of content. This theme was identified in four of the six substantive explanations provided by respondents who were unsure or would not choose medicine again.

One participant who was uncertain about whether they would choose medicine again described a lack of clinical exposure:

“The course I am currently studying lacks the clinical exposure that I would have desired, and I am no longer sure if I am interested in the realities of being a doctor.” (Participant 5)

Another participant described the pace of medical education as a source of frustration:

“It's not been very enjoyable. There have been good moments but mostly just RUSHING through content. Just as i'm [sic] beginning to enjoy something, it's thrown out the window for the next problem.” (Participant 15)

The length and structure of postgraduate training were described by some participants as concerns associated with uncertainty about their future career choice. One participant highlighted the cumulative duration of medical school, foundation training, and subsequent examinations:

“It would take another 5 years to qualify, then a further two years where I would have to rotate around all the hospital specialties, and the exams are never-ending. I am not sure if I have the motivation to complete the degree.” (Participant 5)

Other participants expressed frustration with portfolio requirements and perceived administrative bureaucracy within postgraduate training. One participant described portfolio requirements as “the worst sort of managerial bureaucracy,” characterizing them as a tick-box exercise rather than a meaningful component of professional development.

Job Satisfaction and Future Career Prospects

Some participants compared medicine with alternative careers, both within and outside healthcare. Work-life balance and perceived job satisfaction were recurring considerations. This theme was identified in five of the six substantive explanations provided by respondents who were unsure or would not choose medicine again.

One participant compared medicine with psychology and psychiatry, stating:

“I found that the psychologists were a lot happier with their work-life balances, and even the psychiatrists were unsure if they would pursue a career in medicine again, especially in the NHS right now.” (Participant 5)

One participant who had taken two gap years before entering medical school reported considering alternative careers or leaving the UK, noting that their housemates were considering similar options.

Concerns about postgraduate competition were also prominent. One participant stated:

“I didn't realise how expensive, difficult, and competitive training beyond Foundation would be. The pay does not reflect the extensive training, working conditions, and total working hours each week.” (Participant 9)

The participant subsequently described concerns about competing for postgraduate training while balancing family responsibilities and perceived competition from international graduates.

Another participant expressed concerns regarding increasing competition for specialty training and competition with other healthcare professionals, citing “worsening competition ratios for all specialties” and the perceived loss of opportunities to physician associates and other medical associate professionals.

Inadequate Reward for Effort and Disillusionment With the NHS

Financial considerations were a recurring source of dissatisfaction. Participants described the financial burden of medical education alongside perceptions that doctors' remuneration did not adequately reflect their training and workload. This theme was identified in four of the six substantive explanations provided by respondents who were unsure or would not choose medicine again.

One participant described:

“Terrible pay compared to peers who left school with similar qualifications. Over 100k of student debt with a starting salary lower than at pret a manger [sic].” (Participant 19)

Participants also expressed broader dissatisfaction with the NHS, particularly its working conditions and perceived systemic problems. Some responses suggested that participants had entered medicine with idealized expectations that had subsequently been challenged by their experiences or observations of the healthcare system.

One participant characterized their perception of the NHS as:

“The NHS is crumbling to dust and is currently a toxic race to the bottom.” (Participant 19)

These responses suggested that some participants distinguished dissatisfaction with NHS employment or working conditions from their views of medicine as a profession. Such concerns were described in relation to the organizational and systemic context in which doctors work.

Continued Commitment to Medicine Despite Organizational Dissatisfaction

Participants who would still choose medicine generally described a strong affinity for the profession and a sense that medicine suited them personally. However, this commitment did not necessarily correspond with positive perceptions of the NHS.

One participant stated:

“I love medicine, I love seeing patients and trying to help solve their problems. I just wish I had more control over my life.” (Participant 8)

Another participant described significant dissatisfaction with training conditions and the NHS while nevertheless remaining committed to developing their medical career, stating that they intended to seek better training, pay, conditions, and respect elsewhere.

“I feel very little loyalty to my country and NHS… It’s a pretty rubbish time to be a medical student, particularly as a grad student on an undergrad course. There’s no doubt that I’ll move countries where I’ll get better training, pay, conditions and respect.” (Participant 29)

Within this small free-text subgroup, two respondents who stated that they would still choose medicine again also described negative perceptions of NHS employment, training, or working conditions. These accounts illustrate that stated willingness to choose medicine again could coexist with organizational dissatisfaction. This descriptive finding does not establish a relationship between these perceptions and later career decisions.

The qualitative findings were considered alongside the quantitative results to contextualize observed response patterns, importance-satisfaction discrepancies, and descriptive OccPS and OrgPS scores. Free-text responses illustrated concerns about training, working conditions, remuneration, and future career opportunities. They were not used to test, validate, or establish causal relationships between the quantitative measures and respondents’ stated willingness to choose medicine again. Any subgroup differences in OccPS or OrgPS scores are descriptive only, as no inferential statistical testing was undertaken and the measures are newly developed and unvalidated.

Perceptions of Regret and Potential Solutions

Twelve of the 18 respondents providing free text suggested at least one potential improvement. As suggestions could contribute to more than one category, these categories were not mutually exclusive. Pay or working conditions were raised in six responses; medical education or postgraduate training reform in five; health care system or policy changes in four; and greater priority for UK graduates in training pathways in three. These suggestions represent views expressed within the free-text dataset and should not be interpreted as priorities for UK medical students generally.

The most frequently discussed changes concerned pay and working conditions. Suggestions included restoration of doctors' pay, reduction in working hours, greater control over working patterns, and improved working conditions. Self-rostering was suggested as one potential approach to increasing junior doctors' control over their schedules.

Training reform was another prominent theme. Participants raised concerns about the length and structure of both undergraduate and postgraduate training. Suggestions included restructuring medical education, providing greater exposure to the realities of medical practice, and alternative approaches to training.

Other responses called for broader changes to the health care system and postgraduate workforce structures. These included reducing or ending rotational training, changes to foundation program allocation, greater support for graduate-entry or graduate students on undergraduate courses, and changes to the role and regulation of physician associates and other medical associate professionals.

Finally, some participants expressed concerns about competition for foundation and specialty training positions and argued that UK medical graduates should receive greater priority within training pathways. Several responses linked these concerns to worsening competition ratios and perceived uncertainty regarding access to postgraduate training.

## Discussion

This exploratory mixed-methods study described UK medical students’ stated willingness to choose medicine again alongside occupational perceptions of medicine and organizational perceptions of NHS employment. Fewer than half of participants (45.2%) reported that they would choose medicine again, while 32.3% were unsure and 22.6% would not choose medicine again. Although direct comparisons are limited by differences in populations and measures, career regret or reconsideration has also been reported among medical students in Kazakhstan, Jordan, and India, with stress, the length and demands of medical education, and quality of life identified as important factors [8,10,11]. Similarly, Dyrbye et al. reported an association between characteristics of the medical school learning environment and career regret among United States medical students [9]. The present findings therefore add to the evidence that uncertainty about medicine as a career can emerge before students enter the medical workforce.

A key finding was the distinction between perceptions of medicine itself and perceptions of the NHS. Participants generally retained positive occupational perceptions, with 90.3% agreeing that medicine excited them and 90.3% agreeing that they had the empathy required to be a doctor. In contrast, perceptions of the NHS were considerably less favorable: only 6.5% would recommend working for the NHS, while 77.4% disagreed that the NHS valued its employees and 80.6% disagreed that it was supportive.

Within this sample, positive views of medicine coexisted with negative perceptions of NHS employment and differing career-choice responses; this pattern does not establish whether organizational dissatisfaction contributes to career-choice regret. This interpretation is consistent with UK research identifying working conditions, organizational factors, work-life balance, and training structures among the factors influencing doctors' decisions to leave or reconsider medicine [7,12]. Pathmanathan and Snelling similarly identified lack of control, inadequate support, and work-life balance among important concerns reported by former UK doctors [13].

The importance-satisfaction findings provide further insight into this distinction. Working location, access to specialty training, and work-life balance produced some of the largest discrepancies between what participants considered important and what they expected to obtain from an NHS medical career. Financial factors, including remuneration and the costs associated with training, were also important concerns. These findings broadly complement the discrete-choice experiment by Cleland, in which working conditions were important to UK medical students when considering postgraduate training positions [14]. The prominence of working location in the present study may also reflect contemporary concerns regarding control over training location and postgraduate career progression. However, this interpretation remains tentative and requires investigation in larger samples.

The qualitative findings further supported these quantitative patterns. Participants described concerns about the length and intensity of medical education, examinations and portfolio requirements, competition for postgraduate training, financial pressures, work-life balance, and anticipated working conditions. These concerns overlap with previous UK research identifying workload, working conditions, training opportunities, and work-life balance as important influences on career decision-making among doctors [7,12]. The coexistence of positive attitudes toward clinical medicine with dissatisfaction about training and anticipated NHS employment is particularly notable. It suggests that occupational commitment and organizational dissatisfaction can coexist, potentially producing uncertainty rather than straightforward rejection of a medical career.

Participants also described comparing medicine with alternative careers, particularly in relation to remuneration, working conditions, and the personal costs of medical training. This is consistent with the occupational regret literature, which conceptualizes regret partly in relation to comparisons with alternative choices and perceived outcomes [17]. However, the present study cannot establish whether such comparisons cause regret. Similarly, although most participants (87.1%) considered regret and dissatisfaction among medical students to be common, the higher OccPS and OrgPS scores observed among these participants should be interpreted descriptively given the small, cross-sectional sample.

Overall, the findings describe coexisting stated career-choice responses, occupational perceptions, and organizational perceptions within this small respondent sample. They do not establish a multidimensional structure of career regret, a causal relationship between organizational dissatisfaction and career-choice responses, or implications for future workforce retention. The apparent coexistence of positive views of medicine with negative expectations of NHS employment should be considered a hypothesis for future longitudinal research using validated measures.

Limitations

This study has several limitations. Although the questionnaire remained open for six weeks, the achieved sample was small (n = 31), compared with the planned target of 96. Participants were recruited through convenience and snowball sampling and represented 22 UK universities. The sample may therefore be subject to selection bias and cannot be considered representative of UK medical students. Findings were based on self-reported questionnaire data and may also be affected by response and reporting biases. Findings should consequently be interpreted as exploratory and descriptive rather than as population-level estimates. The cross-sectional design also prevents assessment of changes in perceptions over time and does not permit causal inference.

The OccPS and OrgPS were newly developed measures that had not undergone formal psychometric validation. Although both showed acceptable internal consistency in this sample, as assessed by Cronbach’s alpha, this does not establish construct validity, stability over time, or reliability across populations and settings. In addition, the measures differed in item content; their scores should not be interpreted as directly comparable or as demonstrating distinct validated dimensions of career regret. Findings based on these measures should therefore be interpreted cautiously and as exploratory. Formal psychometric evaluation, including assessment of content and construct validity, factor structure, test-retest reliability, and performance in larger and more diverse samples, is required before the measures can be used as validated measures of occupational or organizational perceptions.

No inferential statistical testing was undertaken, and comparisons between subgroups are descriptive only. The qualitative component was based on brief free-text responses rather than in-depth interviews, limiting contextual depth and interpretation. These limitations are particularly relevant when considering apparent differences between students who would choose medicine again, would not choose medicine again, or were unsure whether they would. The qualitative analysis was conducted by a single researcher using brief free-text responses; no independent coding or formal assessment of coding agreement was undertaken. The themes should therefore be interpreted as an exploratory, descriptive account of recurring patterns within this dataset.

Despite these limitations, this study provides an exploratory description of how respondents viewed medicine, anticipated NHS employment, and reflected on their future career choice. Larger, representative longitudinal studies using formally validated measures are needed to examine how these perceptions develop and whether they are associated with later career decisions.

## Conclusions

Within this small, self-selected exploratory sample of UK medical students, positive views of medicine as a profession coexisted with less favorable expectations of NHS employment and uncertainty about future career choice. Fewer than half of respondents reported that they would choose medicine again, while approximately one-third were unsure and approximately one-fifth reported that they would not. Respondents identified concerns relating to work location, entry into specialty training, work-life balance, the work environment, education and training, career prospects, financial and working conditions, and organizational support. These findings describe coexisting organizational dissatisfaction, career-choice uncertainty, and reports that some respondents would not choose medicine again within this sample; they do not establish that dissatisfaction with the NHS causes career regret or predicts future workforce departure.

Although the occupational and organizational measures showed acceptable internal consistency, they were not validated instruments and differed in content. Their mean scores should therefore not be interpreted as directly comparable or as evidence of a multidimensional structure of regret. The observed relationship between perceptions of medicine, expectations of NHS employment, and career-choice uncertainty should be considered a hypothesis for future research. Larger, representative longitudinal studies using validated measures are needed to examine how these perceptions evolve and whether they are associated with subsequent career decisions.

## Figures and Tables

**Figure 1:**
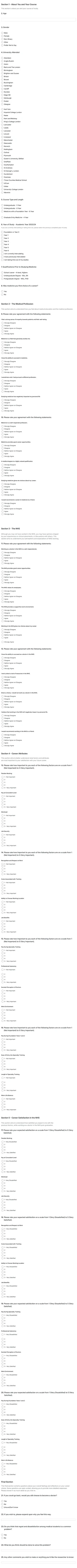
Study questionnaire Image credit: Questionnaire developed by the authors specifically for the present study using Jisc Online Surveys. The figure is an image of the author-created questionnaire exported from the Jisc platform.

## Appendices

Appendix A 

Figure 1 shows the self-administered electronic questionnaire used to collect data for this study. 

Appendix B

Table 6 presents the item-level coding and scoring procedures for the OccPS and OrgPS. 

**Table 6:** Item-level coding for the OccPS and OrgPS All items used the same five-point response anchors: 1 = strongly disagree; 2 = disagree; 3 = neither agree nor disagree; 4 = agree; 5 = strongly agree. Note: Reverse-scored items were recoded before summation using the transformation 1→5, 2→4, 3→3, 4→2, and 5→1. Items were summed to create OccPS and OrgPS total scores, each ranging from 10 to 50. Higher scores indicate more negative occupational perceptions for the OccPS and more negative organizational perceptions for the OrgPS. The OccPS and OrgPS were newly developed, unvalidated exploratory measures and should not be interpreted as validated measures of career regret. OccPS, Occupational Perceptions Scale; OrgPS, Organizational Perceptions Scale

Measure	Item	Questionnaire Statement	Reverse Scored?	Scoring After Recoding
OccPS	Q9_1	I feel a strong sense of empathy towards patients and their well-being.	Yes	Higher score = more negative occupational perception
OccPS	Q9_2	Medicine is a field that genuinely excites me.	Yes	Higher score = more negative occupational perception
OccPS	Q9_3	I have the ability to succeed in medicine.	Yes	Higher score = more negative occupational perception
OccPS	Q9_4	I sometimes wish I had pursued a different profession.	No	Higher score = more negative occupational perception
OccPS	Q9_5	Studying medicine has negatively impacted my personal life.	No	Higher score = more negative occupational perception
OccPS	Q10_1	Medicine is a well-respected profession.	Yes	Higher score = more negative occupational perception
OccPS	Q10_2	Medicine provides good career opportunities.	Yes	Higher score = more negative occupational perception
OccPS	Q10_3	A medical degree is a highly valued qualification.	Yes	Higher score = more negative occupational perception
OccPS	Q10_4	Studying medicine gives me choices about my career.	Yes	Higher score = more negative occupational perception
OccPS	Q10_5	I would recommend a career in medicine to a friend.	Yes	Higher score = more negative occupational perception
OrgPS	Q11_1	Working as a doctor in the NHS is a well-respected job.	Yes	Higher score = more negative organizational perception
OrgPS	Q11_2	The NHS provides good career opportunities.	Yes	Higher score = more negative organizational perception
OrgPS	Q11_3	The NHS values its employees.	Yes	Higher score = more negative organizational perception
OrgPS	Q11_4	The NHS provides a supportive work environment.	Yes	Higher score = more negative organizational perception
OrgPS	Q11_5	Working in the NHS gives me choices about my career.	Yes	Higher score = more negative organizational perception
OrgPS	Q12_1	I have the ability to succeed as a doctor in the NHS.	Yes	Higher score = more negative organizational perception
OrgPS	Q12_2	I worry about a lack of resources in the NHS.	No	Higher score = more negative organizational perception
OrgPS	Q12_3	Given a choice, I would not work as a doctor in the NHS.	No	Higher score = more negative organizational perception
OrgPS	Q12_4	I believe that working in the NHS will negatively impact my personal life.	No	Higher score = more negative organizational perception
OrgPS	Q12_5	I would recommend working in the NHS to a friend.	Yes	Higher score = more negative organizational perception

Appendix C

Table 7 shows the breakdown of participants' responses to each item of the Occupational Perceptions Scale. 

**Table 7:** Participant responses to individual OccPS items (N=31) Source: Questionnaire developed by the authors specifically for the present study. The OccPS was an exploratory, author-developed composite measure. OccPS, Occupational Perceptions Scale

Occupational Perceptions Scale Items		n	%
1. I feel a strong sense of empathy towards patients and their well-being.	Strongly Agree	17	54.8
	Agree	11	35.5
	Neither Agree nor Disagree	1	3.2
	Disagree	2	6.5
2. Medicine is a field that genuinely excites me.	Strongly Agree	17	54.8
	Agree	11	35.5
	Neither Agree nor Disagree	1	3.2
	Disagree	2	6.5
3. I have the ability to succeed in medicine.	Strongly Agree	9	29.0
	Agree	16	51.6
	Neither Agree nor Disagree	3	9.7
	Disagree	3	9.7
4. I sometimes wish I had pursued a different profession.	Strongly Disagree	6	19.4
	Disagree	3	9.7
	Neither Agree nor Disagree	4	12.9
	Agree	8	25.8
	Strongly Agree	10	32.3
5. Studying medicine has negatively impacted my personal life.	Strongly Disagree	3	9.7
	Disagree	3	9.7
	Neither Agree nor Disagree	5	16.1
	Agree	8	25.8
	Strongly Agree	12	38.7
6. Medicine is a well-respected profession.	Strongly Agree	5	16.1
	Agree	13	41.9
	Neither Agree nor Disagree	6	19.4
	Disagree	5	16.1
	Strongly Disagree	2	6.5
7. Medicine provides good career opportunities.	Strongly Agree	4	12.9
	Agree	16	51.6
	Neither Agree nor Disagree	5	16.1
	Disagree	5	16.1
	Strongly Disagree	1	3.2
8. A medical degree is a highly valued qualification.	Strongly Agree	11	35.5
	Agree	14	45.2
	Neither Agree nor Disagree	5	16.1
	Disagree	1	3.2
9. Studying medicine gives me choices about my career.	Strongly Agree	6	19.4
	Agree	11	35.5
	Neither Agree nor Disagree	6	19.4
	Disagree	7	22.6
	Strongly Disagree	1	3.2
10. I would recommend a career in medicine to a friend.	Strongly Agree	4	12.9
	Agree	5	16.1
	Neither Agree nor Disagree	5	16.1
	Disagree	8	25.8
	Strongly Disagree	9	29.0

Appendix D

Table 8 shows the breakdown of participants' responses to each item of the Organizational Perceptions Scale.  

**Table 8:** Participant responses to individual OrgPS items (N=31) Source: Questionnaire developed by the authors specifically for the present study. The OrgPS was an exploratory, author-developed composite measure. OrgPS, Organizational Perceptions Scale

Organizational Perceptions Scale Items		n	%
1. Working as a doctor in the NHS is a well-respected job	Strongly Agree	2	6.5
	Agree	5	16.1
	Neither Agree nor Disagree	8	25.8
	Disagree	9	29.0
	Strongly Disagree	7	22.6
2. The NHS provides good career opportunities	Strongly Agree	2	6.5
	Agree	5	16.1
	Neither Agree nor Disagree	7	22.6
	Disagree	6	19.4
	Strongly Disagree	11	35.5
3. The NHS values its employees	Strongly Agree	1	3.2
	Neither Agree nor Disagree	6	19.4
	Disagree	3	9.7
	Strongly Disagree	21	67.7
4. The NHS provides a supportive work environment	Strongly Agree	1	3.2
	Neither Agree nor Disagree	5	16.1
	Disagree	9	29.0
	Strongly Disagree	16	51.6
5. Working in the NHS gives me choices about my career	Strongly Agree	1	3.2
	Agree	2	6.5
	Neither Agree nor Disagree	11	35.5
	Disagree	7	22.6
	Strongly Disagree	10	32.3
6. I have the ability to succeed as a doctor in the NHS	Strongly Agree	3	9.7
	Agree	14	45.2
	Neither Agree nor Disagree	7	22.6
	Disagree	6	19.4
	Strongly Disagree	1	3.2
7. I worry about a lack of resources in the NHS	Disagree	1	3.2
	Neither Agree nor Disagree	2	6.5
	Agree	5	16.1
	Strongly Agree	23	74.2
8. Given a choice, I would not work as a doctor in the NHS	Strongly Disagree	1	3.2
	Disagree	7	22.6
	Neither Agree nor Disagree	3	9.7
	Agree	6	19.4
	Strongly Agree	14	45.2
9. I believe that working in the NHS will negatively impact my personal life	Strongly Disagree	1	3.2
	Disagree	3	9.7
	Neither Agree nor Disagree	4	12.9
	Agree	3	9.7
	Strongly Agree	20	64.5
10. I would recommend working in the NHS to a friend	Strongly Agree	1	3.2
	Agree	1	3.2
	Neither Agree nor Disagree	3	9.7
	Disagree	10	32.3
	Strongly Disagree	16	51.6
